# Imaging in Periprosthetic Joint Infection Diagnosis: A Comprehensive Review

**DOI:** 10.3390/microorganisms13010010

**Published:** 2024-12-24

**Authors:** Armin Hoveidaei, Yasaman Tavakoli, Mohammad Reza Ramezanpour, Mahyaar Omouri-kharashtomi, Seyed Pouya Taghavi, Amir Human Hoveidaei, Janet D. Conway

**Affiliations:** 1 Exceptional Talents Development Center, Tehran University of Medical Sciences, Tehran 1936893813, Iran; arminhoveidaei@gmail.com; 2 Student Research Committee, Department of Medicine, Mazandaran University of Medical Science, Sari 4815733971, Iran; y.tavakoli@mazums.ac.ir; 3 School of Medicine, Tehran University of Medical Sciences, Tehran 1936893813, Iran; mr.ramezanpour.m@gmail.com; 4 Student Research Committee, Ahvaz Jundishapur University of Medical Sciences, Ahvaz 6135715794, Iran; mahyaar.omouri@gmail.com; 5 Student Research Committee, Kashan University of Medical Sciences, Kashan 8713783976, Iran; taghavi-p@kaums.ac.ir; 6 School of Medicine, Kashan University of Medical Sciences, Kashan 8713783976, Iran; 7 International Center for Limb Lengthening, Rubin Institute for Advanced Orthopedics, Sinai Hospital of Baltimore, Baltimore, MD 21215, USA

**Keywords:** periprosthetic joint infection, imaging, radiological techniques, advanced nuclear medicine techniques

## Abstract

Various imaging methods assist in diagnosing periprosthetic joint infection (PJI). These include radiological techniques such as radiography, computed tomography (CT), magnetic resonance imaging (MRI), and ultrasound (US); as well as advanced nuclear medicine techniques including bone scintigraphy (BS), anti-granulocyte antibody imaging (AGS), leukocyte scintigraphy (LS), and fluorodeoxyglucose positron emission tomography (FDG-PET and FDG-PET/CT). Each imaging technique and radiopharmaceutical has been extensively studied, with unique diagnostic accuracy, limitations, and benefits for PJI diagnosis. This review aims to detail and describe the most commonly used imaging techniques and radiopharmaceuticals for evaluating PJI, focusing particularly on knee and hip arthroplasties.

## 1. Overview

Joint arthroplasty is a highly effective procedure for pain relief and enhancing quality of life. Despite the procedure’s high success rate [1,2], there exists the potential for life-threatening complications such as vascular and nerve injuries, revision surgeries, periprosthetic fractures and dislocations, and periprosthetic joint infection (PJI) [3,4]. Purulence, sinus tract inflammation on histology, or positive cultures are commonly used to define PJI [5]. PJI affects about 2% of people who have had hip and knee arthroplasty and is increasing over time [3]. This complication occurs more frequently in revision arthroplasty, with about 20% of these patients developing PJI [6]. Furthermore, the economic burden of PJI in the United States is projected to reach USD 1.85 billion by 2030 [7].

Preventative measures, such as perioperative antibiotic prophylaxis, aseptic surgical techniques, and optimizing patient factors have been reported to reduce PJI incidence [8]. Antibacterial bone cement, including antibiotic-loaded variants, is widely used for prevention, with novel antibacterial agents currently under investigation to enhance their efficacy [9]. Modifying prosthetic cement properties, such as using faster-setting, stronger, or smoother non-porous materials, is thought to reduce contamination risks, maintain hygiene, and lower infection rates [10,11]. Recent studies have also investigated incorporating admixtures to further improve bone cement properties [12,13,14].

Although advancements in PJI characterization and prevention have been made, the condition persists. This underscores the critical importance of accurate identification to guide effective treatment and improve patient outcomes. However, a consensus on the diagnostic criteria of PJI has yet to be established. Since 2010, respected professional societies have defined PJI using at least seven different sets of criteria [15,16,17,18,19,20,21]. In each of these definitions, various diagnostic techniques have been discussed. However, no single diagnostic method has demonstrated sufficient sensitivity for the accurate diagnosis of PJI [22]. Among these diagnostic approaches, imaging modalities play a limited role despite their potential benefits in infection diagnosis. Previous studies have demonstrated the role of imaging techniques, including computer tomography (CT), magnetic resonance imaging (MRI), and ultrasound (US) in bone and joint infection [23,24]. Moreover, advanced nuclear medicine techniques, such as bone scintigraphy (BS), anti-granulocyte antibody imaging (AGS), leukocyte scintigraphy (LS), and fluorodeoxyglucose positron emission tomography (FDG-PET/CT), can assist in diagnosing suspected PJI [22]. Considering the multiplicity of imaging modalities and the inadequacy of guidelines regarding these methods, physicians face challenges in choosing imaging tools [25].

The most effective imaging modality and radiopharmaceutical are chosen based on how well they work at various phases of the diagnostic procedure [26]. Due to the increasing prevalence of PJI, its clinical and economic burden, and the multiplicity of imaging techniques, there is a need to clarify the role of imaging in PJI diagnosis. This review aims to fill this gap by comprehensively evaluating the diagnostic performance, benefits, and limitations of the most commonly utilized imaging techniques and radiopharmaceuticals. By summarizing the existing evidence, we aim to provide clinicians with a practical guide to selecting the most appropriate imaging modalities for diagnosing PJI in knee and hip arthroplasty.

## 2. Periprosthetic Joint Infection Diagnostic Options

Several diagnostic approaches have been assessed for PJI, but none are accurate enough to confirm PJI on their own. Thus, combining these tests assists in diagnosing the condition. According to EBJIS, these tests can be categorized into six groups: clinical features, serologic tests, synovial fluid analysis, histopathology, microbiology, and imaging methods [21]. Furthermore, a new group of diagnostic approaches includes molecular analysis.

**Clinical Features**: PJI presentation varies based on the infection mechanism, the time of initiation of infection from implantation, pathogen virulence, and host immune response [27,28]. Common signs or symptoms of PJI include pain, joint swelling or fluid accumulation, redness or warmth around the joint, fever, drainage, or the presence of a sinus tract connected to the arthroplasty [29,30]. Moreover, in some PJI definitions, the presence of an abscess or sinus tract is considered a conclusive PJI definitor. Although fever, chills, and joint redness are highly specific indicators, their sensitivity is low [31]. Overall, pain appears to be the most commonly reported clinical symptom [29].

**Serologic Tests**: Erythrocyte sedimentation rate (ESR), C-reactive protein (CRP), interleukin-6 (IL-6), and white blood cell (WBC) count are proposed in the diagnostic criteria for PJI definitions. ESR and CRP are considered first-line serologic tests as they are universally available, inexpensive, and highly sensitive [32]. There is some evidence of increased sensitivity while employing ESR and CRP simultaneously [33]. Although a higher diagnostic odds ratio has been reported for IL-6 assessment compared to ESR and CRP, IL-6 is not as widely available or as cost-effective as ESR and CRP [32,34]. WBC count and polymorphonuclear (PMN) percentage have low sensitivity but show high specificity in the diagnosis of PJI [35].

**Synovial Fluid Analysis**: Synovial fluid, collected via preoperative or intraoperative aspiration, can be used to assess cell count, specific biomarkers, or for bacterial culture [29]. Synovial WBC count and PMN percentage have high sensitivity and specificity. Nevertheless, various cut-off values have been suggested [15,17,18,27]. Evaluating biomarkers such as leukocyte esterase, CRP, IL-6, α-defensin, and D-lactate in synovial fluid can be sensitive or specific enough to assist physicians in diagnosing PJI [27,29,32]. A recent study demonstrated higher accuracy for the combination of PMN% and IL-6 in diagnosing PJI than using PMN% and IL-6 individually [36].

**Histopathology**: Testing the periprosthetic tissue sample can determine acute inflammation. A reported advantage of counting PMNs per high-powered field as one of these tests is that it is unaffected by preoperative antibiotics. However, certain low-virulence organisms, such as *Propionibacterium acnes* or coagulase-negative staphylococci, may not trigger a neutrophilic inflammatory response, which can reduce the sensitivity of the test [27,37,38]. Additionally, intraoperative frozen sections can offer valuable information to surgeons during revision surgery [28].

**Microbiology**: Microbiological tests can identify the organism responsible for the infection. Synovial fluid cultures and periprosthetic tissue cultures can be collected preoperatively and intraoperatively, respectively [28]. According to the definitions of PJI, a diagnosis can be confirmed with at least two culture samples testing positive for the same pathogen. Culture-negative PJIs have a prevalence of 5% to 35% [29]. This condition is largely linked to previous antibiotic therapy. Additional contributing factors involve an inadequate number of samples, extended transport durations, slow-growing microorganisms, and infections caused by mycobacteria, fungi, or fastidious bacteria [27]. The use of sonication to dislodge biofilm from the prosthesis has been suggested in order to increase the sensitivity of periprosthetic tissue culture [39].

**Molecular analysis**: In spite of the fact that molecular examinations have no room in PJI definitions, many studies have assessed their role in PJI diagnosis. Polymerase chain reaction (PCR) and next-generation sequencing (NGS) are two extensively studied methods in the diagnosis of PJI. Both of these methods are beneficial in assessing culture-negative PJIs [40]. However, PCR has certain limitations, including the risk of false-positive results due to DNA contamination, the absence of antimicrobial susceptibility data, and the inability to detect all organisms in polymicrobial infections [41]. Despite the growing use of NGS in microbiology laboratories due to reduced costs and the availability of analytical tools, its most significant challenge remains the complexity of result interpretation [40].

## 3. The Role of Imaging Modalities in PJI Definition Systems

Before 2011, no broadly agreed definition of PJI was available, and diagnosis of this condition mostly relied on deep tissue culture, serologic tests, or manifestation of a sinus tract or abscesses [42]. The first definition of PJI was proposed by the Musculoskeletal Infection Society (MSIS) in 2011 [15]. Following this, the Infectious Disease Society of America (IDSA) in 2013, two International Consensus Meetings (ICM) in 2013 and 2018, MSIS in 2018, the World Association against Infection in Orthopaedics and Trauma (WAIOT) in 2019, and European Bone and Joint Infection Society (EBJIS) defined additional sets of criteria for the diagnosis of PJI [16,17,18,19,20,21] (Table 1). Despite the variety of diagnostic tests, due to their low sensitivity and specificity, there remains a lack of consensus in these definitions, and diagnosing PJI continues to be a challenge [27]. Upon reviewing the seven sets of criteria proposed by various societies, the role of imaging tests in assessing infection appears restricted. Before WAIOT’s definition, imaging techniques had no place in diagnostic criteria. Based on a meta-analysis of the accuracy of nuclear imaging in PJI [43], WAIOT suggested Tc99 bone scan and combined LS and BS as imaging diagnostic techniques for PJI. Later, EBJIS included positive LS as one of the findings that could indicate a likely infection, although in this definition, no imaging test alone can confirm an infection. Considering the valuable reports on imaging modalities assessing bone and joint-related infections and the variety of these tests [44,45], there still seems to be room for incorporating imaging findings into the PJI definition criteria. In light of the sensitivity and specificity of these tests, they can enhance the accuracy of PJI diagnosis when combined with other diagnostic options.

## 4. Advanced Radiological Techniques

### 4.1. Radiography

**Figure 1:**
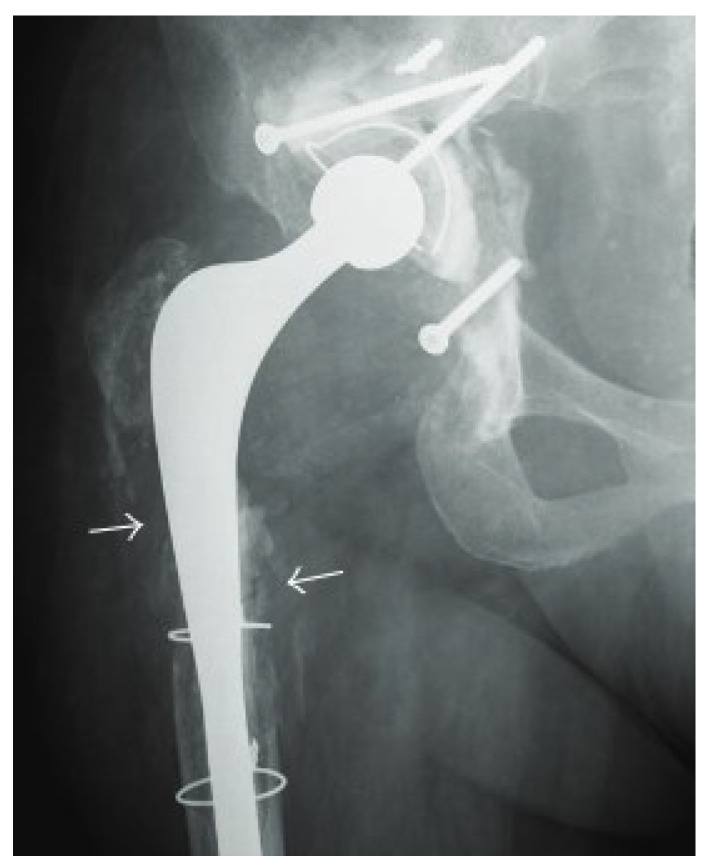
X-ray of PJI after hip arthroplasty; X-ray shows lytic lesions surrounding the femoral component (arrows) [50].

Radiography is the first-line imaging for suspected PJI [46], but its diagnostic utility in post-arthroplasty patients is still debated. Typical radiographic findings of PJI include implant loosening, periosteal reaction, radiolucency at implant interfaces, and osteolysis [47]. Radiographs provide limited evaluation of periprosthetic soft tissues, and useful markers such as interconnecting soft tissue collections are frequently obscured by them. Soft tissue gas, on the other hand, is visible on radiographs when present in sufficient quantities. Soft tissue gas around an arthroplasty is expected in the immediate post-operative period, but its existence beyond 14 days can indicate the possibility of a PJI [48]. The detection of soft-tissue gas on radiography 14 days after total knee arthroplasty (TKA) exhibited a sensitivity of 54% and a specificity of 99% for early PJI, and *Staphylococcus* species were the main organisms [48]. If there is a high clinical suspicion or radiographic indications of infection, image-guided aspiration should be done to confirm the diagnosis [49] (Figure 1).

### 4.2. Ultrasound

Ultrasound (US) has been proven to be a valuable tool in detecting PJI [51], particularly by identifying hypoechoic distention within a prosthetic joint, which may result from synovial thickening, underlying effusion, or a combination of both [52,53]. According to a recent study, a US has a 91% sensitivity for detecting PJI in the hip and knee [54]. A US can differentiate between PJI and aseptic loosening, with extracapsular effusion, joint fluid depth ≥17.0 mm, and grade 2–3 synovial blood flow indicating PJI [55]. A US demonstrates high sensitivity in identifying key PJI features such as joint effusion (70.9%), synovitis (69%), and joint vascularity (67.2%) [56].

**Figure 2:**
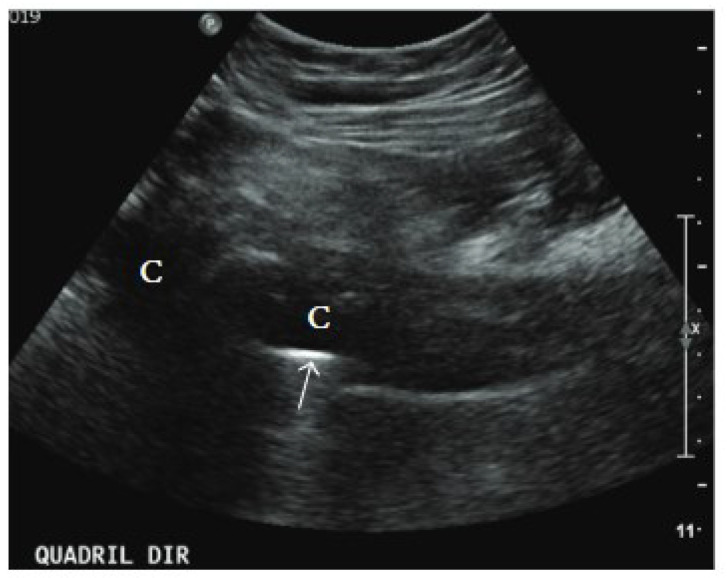
US of PJI after hip arthroplasty; US shows thick fluid collections (C) surrounding the femoral component of the hip prosthesis (arrow) [50].

It has good sensitivity for detecting joint effusions via US-guided arthrocentesis [51]. Two separate investigations on the hip found that US-guided aspiration had a sensitivity of 67% and 69% in detecting PJI [57,58]. While a US is not currently included in established diagnostic algorithms for PJI, its accessibility and non-invasive nature make it a practical first-line option before resorting to more complex procedures [56,59] (Figure 2).

### 4.3. Computed Tomography

Computed tomography (CT) plays an important role in diagnosing PJI by detecting periprosthetic osteolysis and providing superior soft tissue contrast compared to radiography. This enhanced imaging capability improves the identification of periarticular soft tissue abnormalities [60,61]. CT scans are more effective than plain radiography for detecting arthritic changes, bone abnormalities, and cartilage loss after knee arthroplasty. They can also accurately forecast prosthetic sizes and help protect soft tissues during surgery [62]. CT imaging can also be effective in diagnosing hip PJIs, as it reliably detects soft tissue abscesses, joint effusions, and fistulas with high sensitivity and specificity [63,64]. Cyteval et al. found that a CT scan of periprosthetic bone abnormalities did not distinguish between infection and non-sepsis diseases, with the exception of periostitis, which had 100% specificity but only 16% sensitivity. Soft-tissue findings accurately detected infection with 100% sensitivity and 87% specificity. Fluid accumulation in muscles and perimuscular fat exhibited a 100% positive negative predictive value (PPV), but joint distention had a 96% negative predictive value (NPV) [64]. CT can detect soft tissue abscesses and distinguish between septic and aseptic loosening, but metal artifacts can reduce image quality [50,63]. While CT provides advantages such as quick scanning periods, high contrast resolution, and enhanced accuracy, it also has certain drawbacks. The greatest concern is radiation exposure, as CT scans greatly increase annual ionizing radiation exposure [65,66].

**Figure 3:**
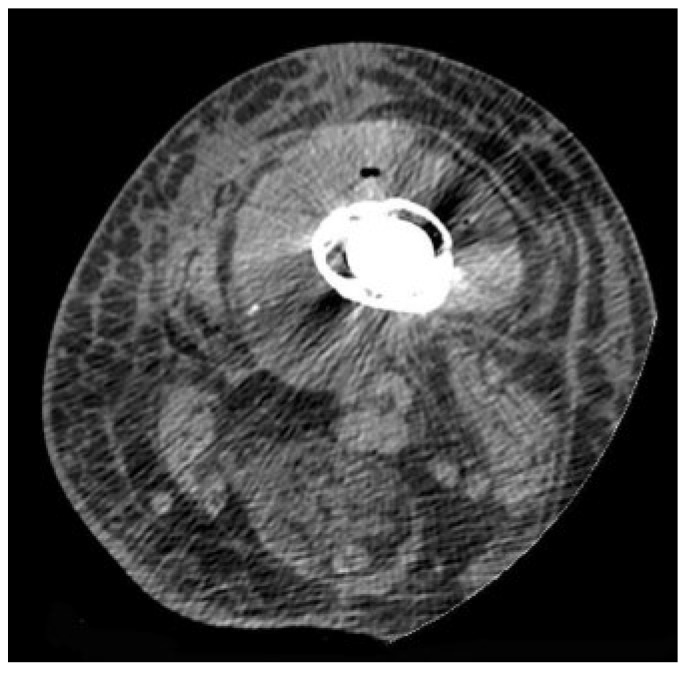
CT scans demonstrate fluid collection and increased density around diseased bone with a prosthetic implant, as well as swelling and hyperdensity of soft tissues due to edema [68].

CT-guided joint aspiration, paired with CT findings, predicted septic hip prosthesis with an accuracy of 86.5%. A substantial volume of aspirated fluid, soft tissue accumulation beyond the joint edge, osteolysis without bone insufflation, and enlarged iliac lymph nodes are all strong indicators of infection [67]. To our knowledge, studies on using CT to diagnose PJI are insufficient, and more research is needed (Figure 3).

### 4.4. Magnetic Resonance Imaging

Magnetic resonance imaging (MRI) provides excellent contrast resolution, enabling detailed evaluation of the prosthesis–bone interface and surrounding soft tissues. MRI can detect bone marrow edema, intramuscular edema, periosteal response, capsule edema, and osteolysis in patients with PJI [69,70]. Other findings suggestive of PJI include joint effusion, capsular thickening, pericapsular edema, and soft-tissue fluid collection [71]. In experimental models, MRI has shown evidence of PJI as early as 1 week post-infection [72]. In 2016, Jiang et al. reported that view angle tilting (VAT) MRI in 86 patients with hip arthroplasty had a sensitivity of 100% in diagnosing soft tissue edema (65), but VAT MRI sensitivity for other PJI characteristics such as soft tissue mass, bone destruction, and fistula was about 50% [73]. Two studies examined lamellated hyperintense synovitis on MRI as a marker of PJI in hip and arthroplasty patients. They reported a sensitivity of 80–90% [74,75]. MRIs with metal artifact reduction approaches have shown great sensitivity in putative PJI indications such as periosteal response (78%), capsule edema (83%), and intramuscular edema (95%) [69]. MRIs generate high-resolution images without radiation exposure, enabling detailed soft-tissue assessment [76]. MRIs have several advantages, including their non-invasive nature, three-dimensional imaging capability, and use of non-toxic contrast chemicals [77]. However, MRIs have limitations, including poor bone visualization and high cost [76]. Other downsides include the potential loss in anatomical imaging contrast due to T1 lengthening and susceptibility effects at high fields [78]. Long scanning intervals, extended breath-hold periods, and motion artifacts might make image interpretation difficult [77]. MRIs are impractical in emergencies and may not effectively detect load-induced instability [76]. However, the quality of evidence supporting MRIs’ function in PJI diagnosis remains low, and there is no agreement on standardized MRI markers for PJI diagnosis. More large-scale investigations using strict protocols are needed to demonstrate MRIs’ diagnostic utility in PJI.

**Figure 4:**
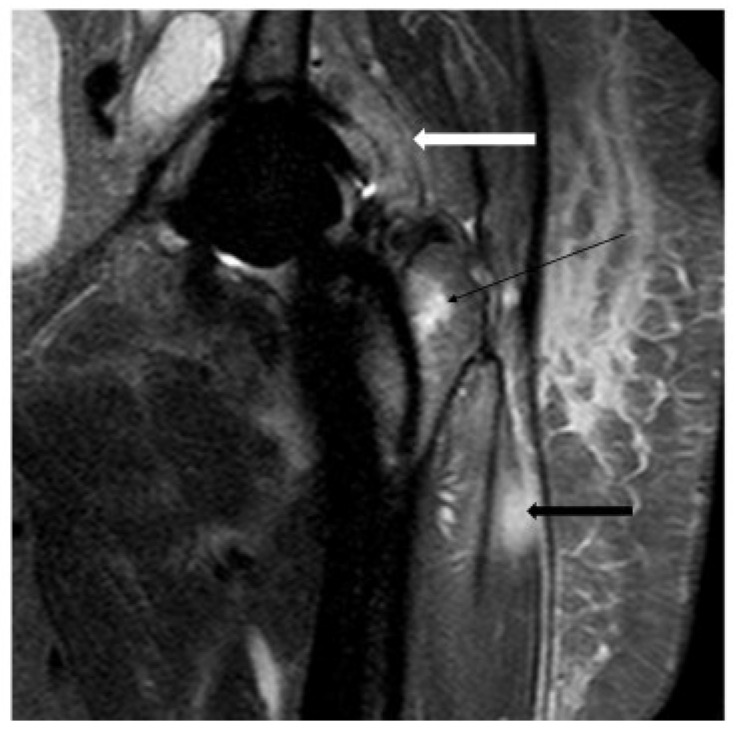
MRI of PJI after hip arthroplasty; MRI imaging reveals layering (white arrow) and synovial hyperintensity, indicating an infection. Femoral bone marrow (a black, thin arrow) and muscle edema (black thick arrow) suggest periprosthetic stress reaction [80].

To our knowledge, as shown in Table 2, six studies have assessed MRIs as an imaging modality in PJI. One study found that MRIs have a sensitivity of 94% and a specificity of 97% for detecting PJI [79]. Another study found that MRIs with metal artifact reduction have a sensitivity of 86% and specificity of 73% in PJI detection [70]. Two studies assessed the MRI detection power of lamellated hyperintense synovitis as a sign of PJI, and MRIs demonstrated a sensitivity of 80% to 92% and a specificity of 84% to 92% in these cases [74,75]. Galley et al. investigated MRIs with metal artifact reduction in the identification of PJI signs, including periosteal reaction, capsule edema, and intramuscular edema, and MRIs exhibited a sensitivity of 78% to 95% and a specificity of 73% to 95%. [69]. A study by Jiang et al. used VAT MRI to detect PJI characteristics such as soft tissue edema, soft tissue mass, bone destruction, and fistula, and a sensitivity of 47.4% to 100%, as well as a specificity of 73.1% to 100%, was granted by using this method [73] (Figure 4).

## 5. Advanced Nuclear Medicine Techniques

### 5.1. Bone Scintigraphy

**Figure 5:**
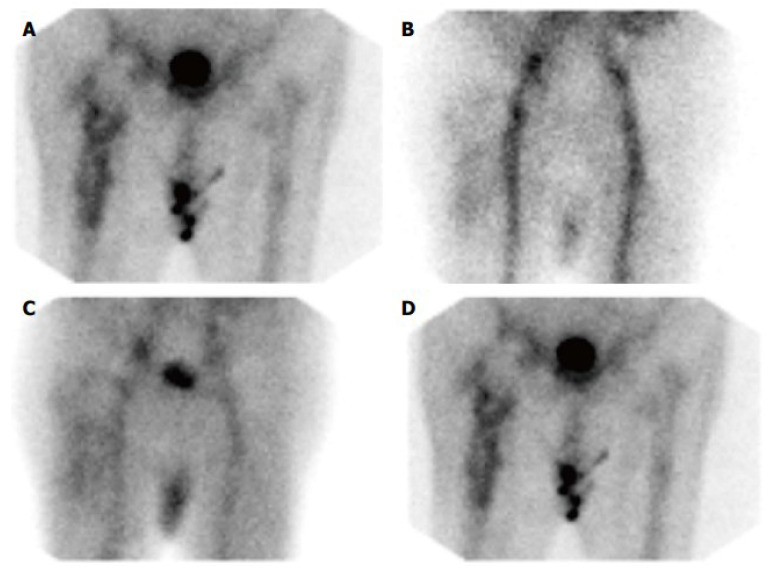
(**A**) BS of PJI after hip arthroplasty. This BS shows irregularly increasing radiopharmaceutical buildup around the femoral component of a prosthesis. (**B**–**D**) On the flow and blood pool pictures, there is diffuse hyperperfusion and hyperemia around the prosthesis, as well as diffusely enhanced periprosthetic radiopharmaceutical on the delayed bone image. (**B**) Flow; (**C**) Blood pool; (**D**) Bone. [87].

Bone scintigraphy (BS) is a diagnostic nuclear tool that mainly uses technetium-99m labeled with phosphate as a radiotracer. Tracers accumulate in sites that have high blood flow and bone remodeling activity, and they reflect their sites on bone scans. Pathological processes like infection and bone metastasis result in increased vascularization and remodeling, making BS a sensitive diagnostic tool [81]. Three-phase bone scan (TPBS) consists of flow, blood pool, and delayed phases, each contributing to diagnosis [82]. According to the consensus paper by the European Association of Nuclear Medicine (ENAM), EBJIS, and the European Society of Radiology (ESR), a negative TPBS rules out PJI. However, a positive TPBS indicates a wide range of pathological and also physiological states and needs further confirmation, along with other modalities like WBC scintigraphy [22]. A meta-analysis of 704 patients evaluated the diagnostic role of TPBS in patients with hip and knee prostheses and reported a sensitivity of 83% and a specificity of 73%. This study indicated TPBS as a suitable screening and diagnostic tool for PJI, especially in hip prostheses, which showed higher sensitivity (81% vs. 75%) and specificity (78% vs. 55%) compared with knee prostheses [83]. A recent meta-analysis in 2017 also indicated BS as a less specific diagnostic tool for PJI in knee prostheses with a specificity of 56% but indicated high sensitivity (93%) for PJI diagnosis [43]. Recent studies further imply the role of BS in ruling out PJI. A study among patients with low-grade PJI without any specific symptoms had a high NPV of 98%, a sensitivity of 71%, and a specificity of 65% when using BS. This study indicated a very low PPV of 8% for the diagnosis of low-grade PJI with BS [84]. Blanc et al. also declared a high sensitivity of 94% for BS in detecting chronic PJI. However, in this study, the specificity and NPV were low (11% and 50%, respectively) compared to the aforementioned studies. Additionally, all the patients had inflammation caused by prosthetic loosening. Apart from infected arthroplasty, BS is often positive because of mechanical loosening and inflammation. Therefore, there was a low specificity and NPV in PJI as a result [85]. The mentioned studies reported a high sensitivity ranging from 71% to 94% and specificity ranging from 11% to 78%. The details of the diagnostic accuracy of BS in PJI can be found in Table 3. BS is highly sensitive for diagnosing PJI, with a negative result effectively excluding the condition. Physicians must be aware that BS should be avoided during the first postoperative year due to likely false positives from normal bone remodeling; LS may provide more reliable results during this period [86] (Figure 5).

### 5.2. Leukocyte Scintigraphy

**Figure 6:**
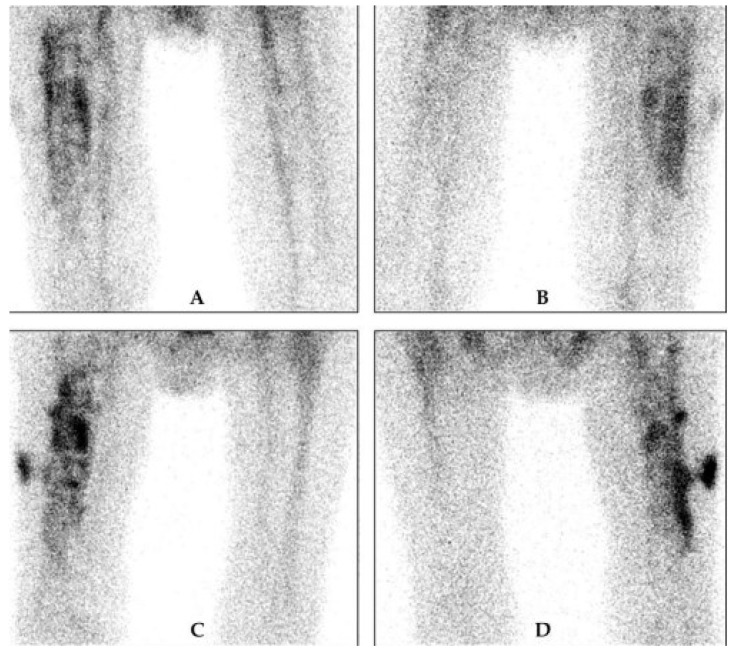
LS of PJI. Delayed images, (**A**) anterior and (**B**) posterior view, late images, (**C**) anterior and (**D**) posterior view. The increase in intensity and size between the delayed and late photos suggests a PJI [68].

Leukocyte scintigraphy (LS) is a type of nuclear medicine that uses autologous WBC, labeled with radiotracers like indium-111 or technetium-99 to locate sites of infection and inflammation [88]. LS is more accurate in detecting PJI compared with BS during the 1st post-surgery year. While a negative BS excludes PJI, a positive result requires confirmation with LS [86]. In a meta-analysis, LS demonstrated a sensitivity of 88% and a specificity of 77% [43]. Recent studies also demonstrated high specificity for LS and introduced it as a specific diagnostic tool in PJI [84,85]. In these studies, LS showed less sensitivity and more specificity compared to BS [43,84,85]. In addition, combining LS with SPECT/CT could improve the diagnosis of PJI, providing a detailed anatomical view of bone and soft tissue involvement and distinguishing between bone marrow accumulation and infected site accumulation of leukocytes. Studies have indicated that adding SPECT/CT to conventional LS enhances both sensitivity and specificity [89,90]. To summarize the diagnostic accuracy of LS in PJI, the sensitivity ranged from 30% to 88% and specificity from 60% to 97%, making it a specific tool for diagnosing PJI. More details of the diagnostic accuracy of LS and LS with SPECT/CT are available in Table 3. LS offers high specificity for PJI diagnosis but is limited by low sensitivity, making it unsuitable as a first-line diagnostic tool. Moreover, LS is a more time-consuming procedure and is not as widely available as bone scintigraphy. Therefore, it is suggested mainly for ruling out infection in cases of positive BS [84] (Figure 6).

### 5.3. Anti-Granulocyte Scintigraphy

Anti-granulocyte scintigraphy (AGS) is a nuclear diagnostic tool that uses a monoclonal antibody that targets leukocyte antigens to identify areas of inflammation [91]. The diagnostic accuracy of AGS has been evaluated for multiple diseases, including osteomyelitis, PJI, vascular prostheses, and inflammatory bowel disease (IBD), and has shown promising results [92]. A meta-analysis investigating the diagnostic role of AGS in PJI in 755 patients reported a pooled sensitivity of 83% (ranging from 57% to 100%), and a pooled specificity of 79% (ranging from 20% to 100%) [93]. An updated systematic review comparing various nuclear modalities in the diagnosis of PJI reported a sensitivity of 90% and a specificity of 95% for AGS. No significant difference was observed regarding sensitivity compared with other nuclear modalities such as BS, LS, and leukocyte scintigraphy-bone marrow scintigraphy (LS/BMS). However, AGS exhibited a significantly higher specificity compared to the other mentioned modalities (specificity for BS, LS, and LS/BMS were 56%, 77%, and 93%, respectively). AGS could be used as an alternative to LS as it is less time-consuming. However, both modalities are not widely available in clinical practice [43]. Blanc et al. evaluated the diagnostic accuracy of different nuclear medicine tools for PJI. Among the included patients, 18 of them underwent AGS. AGS yielded a specificity of 90%, which was superior to the rest of the tests (LS, BS). On the other hand, the use of AGS indicated a sensitivity of 25%, which was significantly lower compared to the other nuclear tools (94% for BS and 72% for LS) [85]. A study from 39 patients with low-grade PJI confirmed that adding SPECT/CT to AGS improved both sensitivity and specificity from 66% to 89% and 60% to 73%, respectively. Combining AGS with SPECT improved the sensitivity (66% to 89%) but failed to improve specificity (60% to 45%) [94]. Altogether, the sensitivity for AGS in detecting PJI varied from 25% to 100%, while the specificity varied from 20% to 95%. The majority of studies reported a sensitivity of over 80% and a specificity above 75% for AGS. The summary of the diagnostic value of AGS is available in Table 3. The sensitivity and specificity of AGS in detecting PJI are promising. However, due to some limitations, including availability, experience in performing the test and interpreting the results, and higher cost compared to BS, it could be limited in use as a confirmation test in patients with positive BS.

### 5.4. Combined Leukocyte and Bone Marrow Scintigraphy

Leukocytes accumulate in both haematopoietically active bone marrow and areas of inflammation. However, sulfur colloid, which is used in bone marrow scintigraphy, only accumulates in bone marrow. Therefore, activity in LS, but not bone marrow scintigraphy, is consistent with infection. Combining these two nuclear method yields additional diagnostic views [95]. A meta-analysis indicated a sensitivity of 80% and specificity of 93% for LS/BMS. LS/BMS significantly outperformed FDG-PET and LS in the detection of prosthetic knee infections. LS/BMS indicated a lower but not significant specificity (93% vs. 95%) and sensitivity (80% vs. 90%) compared with AGS [43]. Combining bone marrow scintigraphy with LS increased the diagnostic value for PJI compared to LS alone [96]. LS/BMS demonstrated great sensitivity (88% and 100%) and specificity (100% and 83%), making it both a sensitive and specific diagnostic modality for the diagnosis of PJI [96,97]. Furthermore, in patients with doubtful LS, additional use of bone marrow scintigraphy yielded a sensitivity and specificity of 84.6% and 93%. Therefore, LS/BMS could be a complementary diagnostic tool in inconclusive LS [98]. As an alternative to LS, AGS could also be combined with BMS and result in greater sensitivity and specificity (100%), which appears to be superior to AGS alone. AGS/BMS may be a viable alternative to LS/BMS, as it is safer and easier to perform. However, more studies with higher participation are needed to achieve a robust conclusion [99]. Basu et al. compared the diagnostic role of LS/BMS with FDG-PET scan in diagnosing PJI. Although LS/BMS indicated a lower sensitivity compared with FDG-PET, it exhibited a favorable specificity (95.7% in hip prostheses and 88.5% in knee prostheses) and was comparable with FDG-PET scans [100]. Similarly, Aleksyniene et al. compared the FDG PET/CT with dual-isotope WBC/bone marrow SPECT/CT in a prospective study, and both modalities exhibited a sensitivity of 100%. However, the dual-isotope WBC/bone marrow SPECT/CT demonstrated higher specificity (97% vs. 71%) [101]. In most studies, sensitivity and specificity for LS/BMS were above 80%, suggesting its diagnostic accuracy. Further details regarding the diagnostic value of LS/BMS in PJI are presented in Table 3. Taken together, LS/BMS is a reasonable imaging method for the detection of PJI with its high sensitivity and specificity. However, it is not widely available, it is costly, and there is a lack of experts to perform and interpret the results. These factors should be considered when using it as a first-line diagnostic tool for PJI.

### 5.5. 18F-Fluorodeoxyglucose Positron Emission Tomography (FDG/PET/CT)

Furthermore, 18F-fluorodeoxyglucose positron emission tomography (FDG/PET) is an advanced imaging technology that visualizes and measures metabolic activity in tissues by labeling a glucose analog with a positron-emitting isotope [102]. FDG/PET is a useful tool in oncology, notably for staging and restaging malignancies, and it is useful in the research of many cancer types, including head and neck, lung, colorectal, and melanoma, although it has limits for other tumors, such as hepatocellular and neuroendocrine carcinoma [103]. FDG-PET is beneficial for detecting musculoskeletal infections and has the potential to diagnose PJI [104,105,106]. The method’s capacity to distinguish between synovitis, loosening, and infection in hip and knee prostheses has been highlighted in the literature [107]. FDG PET/CT integrates PET’s metabolic data with CT’s anatomical detail, providing advantages over PET alone in cancer imaging. This combined technique enables precise localization of enhanced FDG activity, which might be difficult with PET alone [108]. A meta-analysis of 1437 patients found that FDG/PET/CT had a sensitivity of 85%, specificity of 86%, and accuracy of 92% for diagnosing PJI [109]. Another meta-analysis of 635 patients revealed the moderate-to-good overall diagnostic performance of FDG-PET, with a pooled sensitivity of 82.1% and specificity of 86.6% [104]. According to Table 3, whereas all studies revealed a specificity of more than 64% for FDG/PET in PJI diagnosis, two investigations by Kiran et al. found excellent sensitivity (94.87% and 93.75%) but low specificity (<39%) [110,111]. In PJI diagnosis, FDG uptake location is more critical than intensity. Uptake in the femoral component’s center or the bone–prosthesis interface indicates infection [112]. Despite its promise, caution is advised due to heterogeneity and significant false-positive rates of study results [104,110]. Although FDG-PET imaging is a great tool for evaluating many disorders, its high cost and restricted availability are important downsides [113,114].

**Figure 7:**
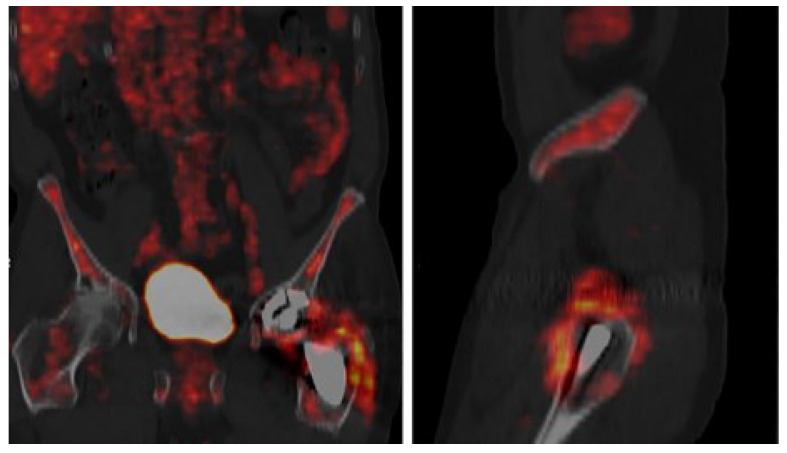
FDG-PET/CT of PJI after hip arthroplasty [68].

To our knowledge, as shown in Table 3, 15 studies have assessed FDG-PET as an imaging modality in PJI and reported a sensitivity of 22% to 100% and specificity of 35.89% to 100% in PJI detection. According to Table 3, three studies have evaluated FDG-PET/CT as an imaging modality in PJI and reported a sensitivity of 85% to 100% and a specificity of 71% to 97.4% in PJI identification (Figure 7).

In Table 4, we summarize the benefits and drawbacks of all vector images described.

**Table 3:** Review of advanced nuclear medicine techniques in PJI diagnosis.

Country	Sample Size/Location of Prosthesis	PJI Definition Criteria	Nuclear Technique	Sensitivity (%)	Specificity (%)	PPV (%)	NPV (%)	Accuracy (%)	References
Multiple (meta-analysis)	499/H	IO with M and H	BS	81	78	NA	NA	NA	[83]
Multiple (meta-analysis)	98/K	IO with M and H	BS	75	55	NA	NA	NA	[83]
Multiple (meta-analysis)	107/K and H	IO with M and H	BS	89	70	NA	NA	NA	[83]
Multiple (meta-analysis)	763/K	IO with C and H	BS	93	56	NA	NA	NA	[43]
France	168/Multi-joint/ (124 for BS)	M	BS	94	11	65	50	64	[85]
The Netherlands	340/K and H	MSIS 2011	TPBS	71	65	8	98	NA	[84]
The Netherlands	142/K and H	MSIS 2011	LS	30	90	25	94	NA	[84]
France	168/Multi-joint (150 for LS)	M	LS	72	60	80	47	67	[85]
Multiple (meta-analysis)	763/K	IO, C and H	LS	88	77	NA	NA	NA	[43]
Spain	105/K and H	M	LS	64	97	78	95	NA	[96]
Turkey	37/ K and H	C and F	LS	100%	59.1%	62.5%	100%	NA	[90]
Turkey	37/K and H	C and F	LS + SPECT/CT	100%	90.1%	88.2%	100%	NA	[90]
Austria	48/Multi-joint	MSIS 2011	LS	60	97	86	90	90	[115]
Korea	71/H	H and M	LS	73.1	93.3	86.4	85.7	85.9	[89]
Korea	93/K	H and M	LS	85.7	80	90	72.7	83.9	[89]
Korea	71/H	H and M	LS + SPECT	80.8	93.3	87.5	89.4	88.7	[89]
Korea	93/K	H and M	LS + SPECT	95.2	80	90.9	88.9	90.3	[89]
Korea	71/H	H and M	LS+ SPECT/CT	88.5	100	100	93.8	88.3	[89]
Korea	93/K	H and M	LS + SPECT/CT	95.2	83.3	92.3	89.3	91.4	[89]
Germany	31/Multi-joint	IO, labs, C, H, and F	AGS	66	60	40	81	NA	[94]
Multiple (meta-analysis)	755/Multi-joint	B, labs, C, H, and F	AGS	83	79	NA	NA	NA	[93]
Multiple (meta-analysis)	763/K	IO, C, and H	AGS	90	95	NA	NA	NA	[43]
France	168/Multi-joint (18 for AGS)	M	AGS	25	90	67	60	61	[85]
Portugal	27/K and H	H and M	AGS	100	20	100	25	NA	[99]
Germany	31/Multi-joint	B, labs, C, H, and F	AGS+ SPECT	89	45	40	91	NA	[94]
Germany	31/Multi-joint	B, labs, C, H, and F	AGS+ SPECT/CT	89	73	57	94	NA	[94]
	56/K and H	IO, H, and M	BMS	84.6	93	NA	NA	91.1	[98]
Spain	105/K and H	M	LS/BMS	88	100	100	89		[96]
USA	59/H	M	LS/BMS	35.5	95.75	71.4	84.6	83.1	[100]
USA	29/K	M	LS/BMS	33.3	88.5	25	92	82.8	[100]
Korea	11/K	IO, H, and M	LS/BMS	100	83	83	100	91	[97]
Multiple (meta-analysis)	763/K	IO, C, and H	LS/BMS	80	93	NA	NA	NA	[43]
Portugal	27/K and H	H and M	AGS/BMS	100	100	100	100	NA	[99]
Denmark	44/K and H	IO and M	LS/BMS SPECT/CT	100	97	93	100	98	[101]
Multiple (meta-analysis)	1437/Multi-joint	IO, H, and C	FDG/PET/CT	85	86	NA	NA	92	[109]
Denmark	48/K and H	IO and M	FDG/PET/CT	100	71	58	100	79	[101]
United Kingdom	130/H	MSIS	FDG/PET	94.87	38.46	60.21	94.59	56.38	[110]
United Kingdom	55/H	C	FDG/PET	93.75	35.89	37.5	93.33	52.7	[111]
USA	87/K	H and M	FDG/PET	94.7	88.2	69.2	98.4	89.7	[100]
USA	134/H	H and M	FDG/PET	81.8	93.1	79.4	94	90.3	[100]
Turkey	46/K and H	H, M, C, and F	FDG/labelled leucocyte PET/CT	93.3	97.4	93.3	97.4	NA	[116]
Germany	32/H	IO and M	FDG/PET	67	83	75	71	NA	[117]
The Netherlands	635/K and H	NA	FDG/PET	82.1	86.6	NA	NA	NA	[104]
USA	113/H	IO, H, and F	FDG/PET	84.9	92.6	80	95	NA	[118]
Spain	24/H	Clinical symptoms, labs, radiography, and joint aspiration	FDG/PET	64.3	64.7	NA	NA	NA	[119]
USA	89/H	NA	FDG/PET	95.2	93	80	98.5	NA	[120]
Germany	27/K and H	IO, H, and M	FDG/PET	40	100	NA	NA	NA	[121]
Germany	63/H	IO and F	FDG/PET	93.9	94.9	NA	NA	95	[122]
Germany	50/H	IO, H, and M	FDG/PET	91	92	NA	NA	91	[123]
Switzerland	35/K	Joint aspiration and F	FDG/PET	R1, 33 R2, 22	R1, 81 R2, 85	NA	NA	R1, 69 R2, 69	[124]
Belgium	17/H	B and F	FDG/PET	87.5	77.8	NA	NA	82.4	[125]
Belgium	21/K	IO and C	FDG/PET	100	73.3	60	NA	NA	[126]
USA	36/K	IO and F	FDG/PET	90.9	72.0	NA	NA	77.8	[105]
USA	38/H	IO and F	FDG/PET	90	89.3	NA	NA	89.5	[105]

Abbreviations: H, hip prosthesis; K, knee prosthesis; NPV, negative predictive value; NA, not available; PPV, positive negative predictive value; C, culture tests; M, microbiological tests; H, histological tests; F, follow-up; IO, intraoperative findings; BS, bone scintigraphy; TPBS, triple-phase BS; LS, leukocyte scintigraphy; AGS, anti-granulocyte scintigraphy; SPECT, single-photon emission computed tomography; FDG, 18F-fluorodeoxyglucose/positron emission tomography; BMS, bone marrow scintigraphy; CT, computed tomography; R, reader.

## 6. Artificial Intelligence-Assisted Imaging for PJI Diagnosis

Artificial intelligence (AI) and machine learning (ML) show promise in predicting and managing PJI following arthroplasty. ML algorithms have shown promising results in predicting PJI and explaining it during implant-based repair, as well as identifying major risk factors for PJI [127]. For TKA, AI tools can assist surgeons in interpreting postoperative X-rays, thereby improving accuracy and sensitivity in detecting anomalies [128]. ML methods, such as support vector machines and neural networks, demonstrate high accuracy in predicting PJI from MRI features. [129,130]. Recent advances in AI offer the potential for predicting PJI based on MRI features as well. Considering bone edema, extracapsular edema, and synovitis as characteristic features of PJI, a support vector machine classifier attained 92% sensitivity and 79% specificity [129]. AI in FDG-PET imaging has the potential to improve the diagnosis and analysis of PJI. AI-based methods can improve detection sensitivity and forecast outcomes in infection and inflammation imaging [131]. These AI-based approaches can aid in patient-specific risk stratification, preoperative counseling, and clinical decision-making. Furthermore, ML has the potential to identify patients at high risk of failure for debridement, antibiotics, and implant retention (DAIR) procedures in acute PJI cases, potentially leading to more tailored treatment strategies [132].

## 7. Future Directions

In this review, we thoroughly discussed the utility of different imaging modalities in diagnosing PJI. Conventional radiological modalities such as MRI, CT scan, and US seemed unsatisfactory. However, studies on these techniques were limited and mainly focused on their effectiveness in detecting PJI features such as edema, effusion, and periprosthetic osteolysis. Further studies should evaluate these widely available imaging tools as potential first-line tools for PJI diagnosis. Considering the increasing interest in AI and ML approaches for their potential to improve medical practice, the lack of studies focusing on this field in diagnosing PJI is notable. ML algorithms incorporating imaging features, clinical manifestations, and laboratory parameters could provide a useful diagnostic perspective in PJI. Advanced nuclear medicine techniques seem more promising and conclusive in diagnosing PJI. However, their diagnostic value varied between studies. For a more robust conclusion, studies comparing different nuclear techniques within a single population with a high number of participants, under the same surgical setting and follow-up strategy, are needed to avoid inconsistencies caused by diverse baseline characteristics across studies. We also observed a lack of studies published in recent years. As imaging techniques are constantly advancing in diagnostic accuracy and level of detail, we hope that researchers will pay more attention to this area and evaluate the ongoing improvements in imaging modalities in diagnosing PJI.

## 8. Conclusions

Imaging techniques currently play a limited role in PJI diagnostic criteria. This study evaluates their utility and effectiveness. Radiography and CT are not recommended for assessing soft tissue and diagnosing PJI. Nevertheless, they may be justified in cases of high clinical suspicion and a lack of other diagnostic options. In contrast, more evidence supports the use of MRI and US, which are more sensitive for detecting soft tissue infections. Because of its widespread availability and relatively straightforward result interpretation, BS is a very sensitive diagnostic technique for diagnosing PJI and might be used as a first-line diagnostic alternative. However, due to the method’s limited specificity, more confirmatory testing is required. LS, AGS, and LS/BMS can be used as supplementary diagnostic modalities because they are more specific and less generally available than BS. Furthermore, LS/BMS improves the accuracy of diagnosis, especially in cases of unclear LS results. Overall, radiological modalities appear to be less effective at diagnosing PJI than modern nuclear medicine procedures. Finally, combining imaging approaches with conventional diagnostic tools has the potential to improve the accuracy of PJI diagnoses.

## Figures and Tables

**Table 1:** Review of different guidelines criteria for PJI diagnosis.

Definition Source	Criteria	Scoring System
**MSIS 2011 [15]**	**Major:** 1. Presence of sinus tract communicating prosthesis 2. Two or more positive cultures for the same pathogen from separate tissue or fluid samples **Minor:** 1. Increased ESR (>30 mm/h) and CRP level (>10 mg/L) 2. Increased synovial WBC count 3. Increased synovial PMN% 4. One positive culture of periprosthetic tissue or fluid 5. >5 PMN in five high-powered fields at ×400 magnification	≥1 major criteria OR ≥4 of 6 minor criteria
**IDSA 2013 [16]**	1. Presence of sinus tract communicating prosthesis 2. Presence of pus around the prosthesis with no other identified cause 3. Acute inflammation observed on histopathological examination of the periprosthetic tissue 4. At least two positive intraoperative cultures OR the same pathogen in preoperative aspiration culture and intraoperative culture OR one positive culture of a highly virulent microorganism	≥1 positive criteria
**ICM 2013 [17]**	**Major:** Same as the major criteria of MSIS 2011 **Minor:** 1. Increased ESR (>30 mm/h) and CRP level (acute infection: >100 mg/L; chronic infection: >10 mg/L) 2. Increased synovial fluid WBC count (acute infection: >10,000 cells/mL; chronic infection: >3000 cells/mL) OR ++ result in leukocyte esterase test strip 3. Increased PMN% (acute infection: >90%; chronic infection: >80%) 4. >5 PMN in five high-powered fields at ×400 magnification 5. One positive culture	≥1 major criteria OR ≥3 of 5 minor criteria
**ICM 2018 [20]**	**Major:** Same as the major criteria of MSIS 2011 **Minor:** (a) Increased CRP level (acute infection: >100 mg/L; chronic infection: >10 mg/L) OR D-dimer level (chronic infection: >860 ug/L; unknown cut-off for acute infection): score 2 (b) Increased ESR (chronic infection: >30 mm/h; no role in acute infection): score 1 (c) Increased synovial WBC count (acute infection: >10,000 cells/mL; chronic infection: >3000 cells/mL) OR ++ result in leukocyte esterase test OR positive α-defensin test: score 3 (d) Increased synovial PMN% (acute infection: >90%; chronic infection: >70%): score 2 (e) One positive culture: score 2 (f) Positive histology: score 3 (g) Positive intraoperative pus presence: score 3	≥1 major criteria: infected OR minor scoring criteria: ≥6 infected 3–5 inconclusive <3 not infected
**MSIS 2018 [18]**	**Major:** Same as the major criteria of MSIS 2011 **Minor preoperative:** (a) Increased CRP OR D-Dimer level in serum: score 2 (b) Increased ESR in serum: score 1 (c) Increased synovial WBC count OR leukocyte esterase: score 3 (d) Positive α-defensin test in synovial fluid: score 3 (e) Increased synovial PMN%: score 2 (f) Increased synovial CRP level: score 1 **Minor intraoperative:** (a) Positive histology: score 3 (b) Positive purulence: score 3 (c) One positive culture: score 3	≥1 major criteria: infected OR Minor preoperative scoring criteria: ≥6 infected 2–5 possibly infected <2 not infected OR Minor intraoperative scoring criteria: ≥6 infected 4–5 inconclusive ≤3 not infected
**WAIOT [19]**	**Rule OUT tests:** each negative test score is −1, and positive test scores are 0 (a) ESR > 30 mm/h (b) CRP > 10 mg/L (c) WBC > 1500/µL (d) leukocyte esterase ++ (e) α-defensin > 5.2 mg/L (f) Tc99 bone scan **Rule IN tests:** each positive test score is +1, and negative test scores are 0 (a) Presence of pus or draining sinus or exposed joint prosthesis (b) Serum IL-6 > 10 pg/mL (c) Serum Procalcitonin > 0.5 ng/mL (d) Serum D-Dimer >850 ng/mL (e) Synovial fluid cultural examination (f) Synovial fluid WBC count > 3000/mL (g) Leukocyte esterase ++ (h) Synovial fluid α-defensin > 5.2 mg/L (i) Combined leukocyte and bone marrow scintigraphy (j) Frozen section 5 PMN in at least 3 high-powered fields	<0 score/one or more condition(s), other than infection, can cause the symptoms/negative cultural examination: **no infection** <0 score/one or more condition(s), other than infection, can cause the symptoms/one positive culture with negative histology: **contamination** <0 score/“unexplained” pain OR swelling OR stiffness/positive cultural examination OR positive histology: **biofilm-related implant malfunction** ≥0 score/pain OR swelling OR stiffness/positive cultural examination OR positive histology: **low-grade PJI** ≥1 score/two or more of pain, swelling, redness, warmth, *functio laesa/*positive cultural examination OR positive histology: **high-grade PJI**
**EBJIS [21]**	**Infection likely criteria:** 1. Radiological evidence of implant loosening occurring in the first 5 years 2. History of complications with wound healing 3. Recent history of fever or bacteremia 4. Presence of pus surrounding the prosthesis 5. Serum CRP > 10 mg/L 6. Synovial fluid WBC count > 1500 7. Synovial fluid PMN% > 65% 8. Positive culture of aspiration fluid 9. Intraoperative specimen single positive culture 10. >1 CFU/mL of any organism in sonication 11. ≥5 PMNs in a single high-powered field 12. Positive leukocyte scintigraphy **Infection confirmed criteria:** 1. Presence of sinus tract communicating the joint or exposing the joint prosthesis 2. Synovial fluid WBC count > 3000 3. Synovial fluid PMN% > 80% 4. Synovial fluid positive α-defensin 5. At least two intraoperative culture samples testing positive for the same microorganism 6. >50 CFU/mL of any organism in sonication 7. ≥5 PMNs in a ≥5 high-powered field 8. Presence of visible microorganisms	Two positive findings: **infection likely** (only if there is a positive clinical feature or raised serum CRP) Any positive finding: **infection confirmed**

Abbreviations: MSIS, the Musculoskeletal Infection Society; IDSA, Infectious Diseases Society of America; ICM, International Consensus Meeting; WAIOT, World Association against Infection in Orthopedics and Trauma; EBJIS, European Bone and Joint Infection Society; ESR, erythrocyte sedimentation rate; CRP, C-reactive protein; WBC, white blood cell; PMN, polymorphonuclear; IL-6, interleukin-6; PJI, periprosthetic joint infection.

**Table 2:** Review of radiological techniques in PJI diagnosis.

Country	Sample Size/Prostheses Location	PJI Definition Criteria or Diagnostic Variables	Radiologic Technique	Sensitivity (%)	Specificity (%)	PPV (%)	NPV (%)	Accuracy (%)	References
USA	15/K	NA	Radiography	54	99	NA	NA	NA	[48]
France	54/K and H	MSIS	US	91	19	64	57	NA	[54]
Egypt	70/multi-joint	Joint effusion	US	70.9	100	NA	NA	62.8	[56]
Egypt	70/multi-joint	Synovitis	US	69	83.4	NA	NA	71.4	[56]
Egypt	70/multi-joint	Erosions and bone lesions	US	50	50	NA	NA	50	[56]
Egypt	70/multi-joint	Soft tissue affection	US	56.4	75	NA	NA	58.6	[56]
Egypt	70/multi-joint	Joint vascularity	US	67.2	100	NA	NA	74.3	[56]
Italy	60/H	C	US-guided aspiration	69	94	NA	NA	83	[57]
Norway	80/H	NA	US-guided biopsy specimens	67	68	22	94	NA	[58]
Spain	96/H	M and C	CT-guided joint aspiration	NA	NA	NA	NA	86.5	[67]
France	65/H	At least one soft tissue abnormality was used as an infection criterion	CT	100	87	NA	NA	89	[64]
France	65/H	Joint distention as an infection criterion	CT	83	96	NA	NA	94	[64]
Switzerland	40/H	Periosteal reaction	MRI with metal artifact reduction	78	90	NA	NA	86	[69]
Switzerland	40/H	Capsule edema	MRI with metal artifact reduction	83	95	NA	NA	91	[69]
Switzerland	40/H	Intramuscular edema	MRI with metal artifact reduction	95	86	NA	NA	89	[69]
Germany	41/H	Clinical and IO	MRI with metal artifact reduction	86	73	NA	NA	NA	[70]
China	50/H	Lamellated hyperintense synovitis	MRI	80–88	84–92	83–92	81–88	NA	[75]
China	86/H	Soft tissue mass	VAT MRI	52.6	89.6	NA	NA	NA	[73]
China	86/H	Soft tissue edema	VAT MRI	100	73.1	NA	NA	NA	[73]
China	86/H	Bone destruction	VAT MRI	47.4	92.5	NA	NA	NA	[73]
China	86/H	Fistula	VAT MRI	47.4	100	NA	NA	NA	[73]
China	56/H	Intraoperative findings with M and H	MRI	94	97	NA	NA	NA	[79]
USA	28/K	Lamellated hyperintense synovitis	MRI	86–92	85–87	NA	NA	NA	[74]

Abbreviations: H, hip prosthesis; K, knee prosthesis; NPV, negative predictive value; NA, not available; PPV, positive predictive value; US, ultrasonography; MRI, magnetic resonance imaging; CT, computed tomography; VAT, view angle tilting; C, culture tests; M, microbiological tests; H, histological tests; IO, intraoperative findings.

**Table 4:** Strengths and limitations of radiological and advanced nuclear medicine techniques in PJI diagnosis.

Imaging Technique	Strengths	Weaknesses
Computed tomography (CT)	Higher soft tissue contrast compared to radiography High specificity for certain features Can be used to guide diagnostic procedures like aspiration Wide availability and moderate cost	Normal appearance in initial infection May be degraded by metal artifacts of prostheses Limited sensitivity
Magnetic resonance imaging (MRI)	Early detection of PJI in experimental models Comprehensive examination of PJI characteristics Wide availability and no radiation exposure	Low quality of evidence regarding MRI Metal artifacts
Ultrasound	High sensitivity Guidance for arthrocentesis Non-invasive and accessible	Operator dependency
Bone scintigraphy	High sensitivity and NPV Effective as a screening test, especially in hip arthroplasty Wide availability	Low specificity and PPV Limited use post-surgery Need for additional tests for further confirmation
Anti-granulocyte-antibody scintigraphy	High sensitivity and specificity Less time-consuming than leukocyte scintigraphy Potential as an alternative test for leukocyte scintigraphy Enhanced diagnostic accuracy when combined with SPECT/CT	Moderate PPV and NPV Limited availability
Leukocyte scintigraphy	High specificity and NPV Useful in early post-surgery period Enhanced diagnostic accuracy when combined with SPECT/CT	Low sensitivity and PPV Time-consuming and less available than bone scintigraphy Varied diagnostic performance in different studies
Combined WBC and bone marrow scintigraphy	Increased diagnostic accuracy Useful in doubtful leukocyte scintigraphy Better differentiation between infection and bone marrow activity	Time and resource intensive Not widely available
F-Fluorodeoxyglucose positron emission tomography (FDG-PET/CT)	High sensitivity and specificity Differentiation between PJI characteristics	Heterogeneity of study results and significant false-positive rates Interpretation challenges Poor availability and high cost

Abbreviations: PJI, periprosthetic joint infection; PPV, positive predictive value; NPV, negative predictive value.

## Data Availability

No new data were created or analyzed in this study. Data sharing is not applicable.
